# Synthetic Lesions with a Fluorescein Carbamoyl Group As Analogs of Bulky Lesions Removable by Nucleotide Excision Repair: A Comparative Study on Properties

**DOI:** 10.32607/actanaturae.27419

**Published:** 2024

**Authors:** A. A. Popov, V. M. Golyshev, L. S. Koroleva, K. D. Nazarov, R. O. Anarbaev, I. O. Petruseva

**Affiliations:** Institute of Chemical Biology and Fundamental Medicine, Siberian Branch of Russian Academy of Sciences, Novosibirsk, 630090 Russian Federation

**Keywords:** nucleotide excision repair, bulky lesion, spectrometric titration

## Abstract

Mammalian nucleotide excision repair (NER), known for its broad substrate specificity, is responsible for removal of bulky lesions from DNA. Over 30 proteins are involved in NER, which includes two distinct pathways: global genome NER and transcription-coupled repair. The complexity of these processes, the use of extended DNA substrates, and the presence of bulky DNA lesions induced by chemotherapy have driven researchers to seek more effective methods by which to assess NER activity, as well as to develop model DNAs that serve as efficient substrates for studying lesion removal. In this work, we conducted a comparative analysis of model DNAs containing bulky lesions. One of these lesions, N-[6-{5(6)-fluoresceinylcarbamoyl}hexanoyl]-3-amino-1,2-propanediol (nFluL), is known to be efficiently recognized and excised by NER. The second lesion, N-[6-{5(6)-fluoresceinylcarbamoyl}]-3-amino-1,2-propanediol (nFluS), has not previously been tested as a substrate for NER. To evaluate the efficiency of lesion excision, a 3’-terminal labeling method was employed to analyze the excision products. The results showed that nFluS is removed approximately twice as efficiently as nFluL. Comparative analyses of the effects of nFluL and nFluS on the geometry and thermal stability of DNA duplexes — combined with spectrophotometric and spectrofluorimetric titrations of these DNAs with complementary strands — were performed next. They revealed that the absence of an extended flexible linker in nFluS alters the interaction of the bulky fluorescein moiety with neighboring nitrogenous bases in double-stranded DNA. This absence is associated with the enhanced efficiency of excision of nFluS, making it a more effective synthetic analog for studying bulky-lesion removal in model DNA substrates.

## INTRODUCTION

The integrity and stability of the genome are maintained by DNA repair mechanisms. One such mechanism is nucleotide excision repair (NER), which is responsible for the removal of bulky lesions. These lesions are typically covalent adducts that introduce significant changes into the regular structure of DNA. NER is a multistep process during which proteins sequentially assemble multisubunit complexes of variable composition at a site of DNA damage. The damage is then recognized and excised, along with a surrounding segment of DNA, typically 24 to 32 nucleotides in length. The original DNA sequence is restored by repair polymerases and ligases, using the intact strand as a template. There are two branches of NER: global genome NER (GG-NER), which operates independently of the transcriptional activity of the genome, and transcription-coupled repair [1, 2].

The NER system is characterized by broad substrate specificity and is capable of removing a variety of DNA lesions. These include damage caused by UV and ionizing radiation or by chemically active environmental substances like polycyclic aromatic hydrocarbons and their reactive metabolites (e.g., diol epoxides). The mechanism of action of many chemotherapeutic drugs also involves the formation of bulky DNA adducts. Numerous studies have been conducted to investigate the NER mechanism. The major proteins involved in NER have been identified, along with their roles, interactions, and key stages of the repair performed by them [1, 3]. Nonetheless, many details of the NER mechanism, including the specific roles of certain proteins, remain unclear and are still being explored at the level of protein–nucleic-acid complexes [4, 5, 6, 7]. A key area of interest, both in basic and applied research, is the comparative assessment of NER activity *in vitro*. In such studies, GG-NER activity is typically evaluated using model substrates: linear DNA duplexes at least 120 base pairs (bp) long, with a bulky lesion introduced into one strand at an internal position [8, 9, 10, 11]. Similar DNA duplexes of various lengths are also utilized to study the interactions of damaged DNA with the recombinant proteins involved in both NER branches [7, 12], as well as to examine NER activity in the context of nucleosomes [4].

Model DNAs are frequently constructed using synthetic analogs of lesions, introduced into DNA via automated synthesis. This process imposes specific requirements on the properties of the modified nucleotide incorporated into a DNA strand [13]. Finding synthetic analogs of bulky DNA lesions that can be i) effectively removed by the NER system and ii) stably and efficiently incorporated into model DNAs remains an important pursuit in this field. These model DNAs can be used to assess NER activity, including practical applications such as determining the resistance of cells to chemotherapy-induced or spontaneous DNA damage. These DNAs also serve as valuable tools for studying in detail the mechanisms of NER.

**Fig. 1:**
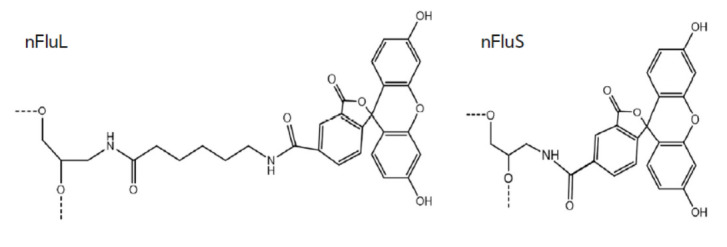
Synthetic analogs of the lesions used in this work. nFluL – N-[6-{5(6)-fluoresceinylcarbamoyl}- hexanoyl]-3-amino- 1,2-propanediol; nFluS – N-[6-{5(6)-fluoresceinylcarbamoyl}]- 3-amino-1,2-propanediol

In this work, we conducted a comparative analysis of model DNA duplexes containing one of two synthetic bulky lesions that differ in the way the N-[6-{5(6)-fluoresceinylcarbamoyl}] group is attached to a propanediol moiety. By means of DNA containing N-[6-{5(6)-fluoresceinylcarbamoyl}hexanoyl]- 3-amino-1,2-propanediol (nFluL), which is effectively removed by NER proteins, we earlier performed comparative analyses of NER activity in cell extracts from various mammals [14, 15, 16, 17]. N-[6-{5(6)- fluoresceinylcarbamoyl}]-3-amino-1,2-propanediol (nFluS) as a model lesion has not been researched previously (*Fig. 1*).

The structure of the nFluS lesion, which lacks a hexanoyl linker, was selected based on our studies on the efficiency of initial recognition of lesion-containing DNA regions by XPC protein complexes. This recognition involves detecting a bulky lesion in the destabilized double-stranded DNA (dsDNA) region by ATPdependent 5’→3’ helicase XPD, a subunit of the TFIIH complex. By analyzing several model DNAs containing lesions that are excised at various efficiency rates by the NER system, we previously demonstrated that XPD exhibits the strongest affinity for an nFlu-containing model DNA [18]. Molecular dynamics simulations indicate that the presence of an extended flexible linker allows the fluorescein moiety to shift its position and interact with multiple regions of dsDNA surrounding the lesion, including the 3’ side, thereby leading to the destabilization of the DNA structure in this region [17, 18]. XPC binding to this destabilized DNA region results in the formation of repair-unproductive XPC–DNA complexes, because the TFIIH verification complex, which binds to the 3’ side of the lesion and moves in the 5’→3’ direction, fails to encounter the lesion and dissociates from the DNA [19, 20]. Although NMR methods afford us the most detailed elucidation of the structure and interactions of bulky DNA lesions, these techniques require milligram quantities of DNA samples containing a lesion.

Here, in addition to the comparative evaluation of the efficiency of excision of nFluS and nFluL by proteins from a NER-competent HeLa cell extract, we also assessed the influence of nFluS or nFluL at an internal position of one strand of a 16 bp DNA duplex on dsDNA geometry and thermal stability. This was done by thermal denaturation assays with optical signal detection. Spectrometric titration experiments were also conducted, yielding data on the interaction of the polycyclic moieties of the bulky lesions with neighboring nitrogenous bases in DNA [21].

## EXPERIMENTAL

**Materials**

An NER-competent extract from HeLa cells was prepared by a standard protocol [22]. T4 polynucleotide kinase, T4 DNA ligase, and Taq DNA polymerase were acquired from Biosan (Russia), whereas proteinase K, EDTA, Tris, HEPES, and dithiothreitol (DTT) came from Sigma (USA). ODNs were synthesized in the Laboratory of Biomedical Chemistry, Institute of Chemical Biology and Fundamental Medicine, SB RAS. The amidophosphites used in the synthesis were provided by NanoTech-S (Russia). [γ-32P] ATP (3000 Ci/mmol) and [α-32P]dCTP (3000 Ci/mmol) were obtained from the Institute of Chemical Biology and Fundamental Medicine, SB RAS. The following materials were also employed: DEAE filters DE-81 (Whatman, UK), urea, N,N′-methylenebisacrylamide (Amresco, USA), acrylamide (Applichem, Germany), and TEMED (Helicon, Russia). Other chemicals used included PSA, MgCl_2_, NaCl, H_3_BO_3_, NaOH, sodium cacodylate, LiClO_4_, (NH_4_)_2_SO_4_, HCl, and acetone.

**Table 1:** Oligodeoxyribonucleotides (ODNs) used in this work

No.	Sequence	Length, nt.	Description
1	P-5′-atccagggcgacggtg	16	Unmodified strand (middle link)
2	P-5′-atccagggmSgacggtg	16	ODN with a non-nucleotide unit carrying a fluorescein residue (nFluS) for incorporation into the upper strand (middle unit, ODN-2)
3	P-5′-atccagggmLgacggtg	16	ODN with a non-nucleotide unit carrying a fluorescein residue and with a linker based on aminohexanoic acid (nFluL), for incorporation into the upper strand (middle unit of the upper strand, ODN-3)
4	P-5′-caccgtcgccctggat	16	A lower strand without a modification
5	5′-tggacgatatcccgcaagaggcccggcagtaccggcataaccaagcctatgcctacagc	59	A 5′ component of the top strand (the left arm of the top strand)
6	P-5′-ccgaggatgacgatgagcgcattgttagatttcatacacggtgcctgactgcgttagcaatt	62	A 3′ component for the top strand (the right arm of the top strand)
7	5′-catcctcggcaccgtcgccctggatgctgtaggcatag	38	A complementary strand for ligation of three fragments of the upper strand of the base sequence
8	5′- tgcgctcatcgtcatcctcggcaccgtcgccctggatgctgtaggcataggctt	54	A lower unmodified strand (middle link for the lower strand, ODN-7)
9	5′-gggggcgtaccttgtgagcaatcgtgttcatcat-P	34	A template for elongation (completion) of excision products [α-³²P]dCMP
10	5′-P-ggttatgccggtactgccgggcctcttgcgggatatcgtcca	42	A 3′ component for the lower strand (the right arm of the lower strand)
11	5′-cgatgagcgcattgttagatttc	23	The complementary strand for ligation of ODN-7 and the left arm of the lower strand
12	5′-agtaccggcataaccaagcctatgcc	26	The complementary strand for ligation of ODN-7 and the right arm of the lower strand

The nucleotide sequences of all the ODNs utilized in this work are listed in *Table 1*.

**Synthesis of extended model DNAs**

Long model DNAs (137 bp) were constructed from ODNs 1–3 and 5–12 with the help of T4 DNA ligase, as previously described. The sequences of the ODNs and the extended model DNAs were identical to those used in our earlier studies [14].

**Evaluation of the substrate properties of model DNA duplexes containing fluorescein adducts**

For a comparative analysis of specific excision efficiency, the 3’-end–labeling method for excision products was employed [14]. The reaction mixture (30 μl), which contained 16 nM DNA substrate, 1.6 mg/ml NER-competent cell extract, and 0.5 μM template ODN-9 for hybridization of excision products in a buffer (25 mM Tris-HCl pH 7.8, 45 mM NaCl, 4.4 mM MgCl_2_, 0.1 mM EDTA, and 4 mM ATP), was incubated at 30°C for 10–0 min. The reaction was inactivated by heating the mixture to 95°C, followed by cooling to room temperature. After the addition of 3 μl of a mixture containing 100 μM dATP, dGTP, and dTTP, 5 units of Taq DNA polymerase, and 500–750 Bq [α-32P]dCTP, the reaction was incubated at 37°C for 5 min. Next, 0.5 μl of 50 μM dCTP was added and the reaction was incubated for an additional 15 min. The reaction was terminated by the addition of solutions of proteinase K (4 μg/ml) and 10% SDS (1 μl each), followed by incubation for 30 min at 37°C. The reaction products were precipitated with 96% ethanol, centrifuged at 12,000´ *g* (4°C), and washed with 70% ethanol, and the resulting pellet was dissolved in water. The reaction products were separated by denaturing polyacrylamide gel electrophoresis (PAGE). Gels containing radioactively labeled DNA samples were analyzed using an Imaging Screen-K radioluminescent screen (Kodak, USA), followed by scanning on a GE Typhoon FLA 9500 instrument. Quantitative analysis of the results was performed in the Quantity One software.

**Determination the bending angle of DNA duplexes**

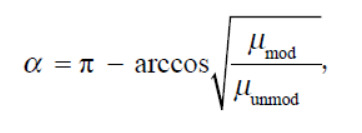



where μ_mod_ and μ_unmod_ are the electrophoretic mobility rates of duplexes during separation in a nondenaturing gel.

**Evaluation of the DNA duplexes’ thermal stability by thermal denaturation with optical signal recording**

The thermal stability of the DNA duplexes was evaluated on a Cary 300-Bio spectrophotometer (Varian, Australia) equipped with a six-section Peltier element for temperature variation and quartz cuvettes with a 0.2 cm optical path length. The temperature range was set between 5°C and 95°C. Sample temperatures in each cuvette were calibrated using Temperature Probes Series II thermocouples (Varian, Australia). Measurements were taken at wavelengths of 260, 270, and 300 nm (baseline; slit width: 1 nm, signal averaging time: 1 s, temperature change rate: 0.5°C/min). All thermal denaturation samples were dissolved in Mili-Q deionized water and 10 mM cacodylate buffer [(CH_3_)2AsO_2_Na] pH 7.2 containing 0.1 M NaCl. Thermodynamic parameters were calculated via analysis of changes in the optical density during heating and cooling at wavelengths of 260 and 270 nm, with theoretical curve fitting based on a two-state model in the Simplex software. Data characterizing the DNA thermal stability were stored in Microsoft Excel.

**Spectrometric titration**

This procedure was performed using 16-mer ODNs (*Table 1*). Two series of samples containing ODN- 2 (nFluS-ODN) or ODN-3 (nFluL-ODN) at a constant concentration (5.4 μM) and a complementary strand (ODN-4) at various concentrations, from 1.25 to 5.4 μM, were prepared in 20 mM Tris-HCl buffer pH 8.0, containing 50 mM NaCl. The control samples contained only an ODN carrying a modification. Samples were incubated for 5 min at 95°C in 1× TE buffer, followed by cooling at 1°C/min to room temperature to enable duplex formation. Spectra were recorded on a CLARIOstar plate reader spectrofluorimeter (BMG Labtech, Germany) in Corning 3635 UV-transparent plates. The standard deviation of the data was calculated via the formula:

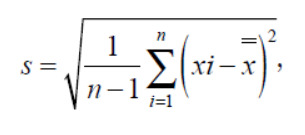



where *n *is the sample size, *xi *is the *i*th element, and =*x *is the sample mean. The error bars in all figures represent standard deviations based on at least three independent experiments.

## RESULTS AND DISCUSSION

**Comparative evaluation of the efficiency of nFluL and nFluS removal from model DNAs in the specific excision reaction**

**Fig. 2:**
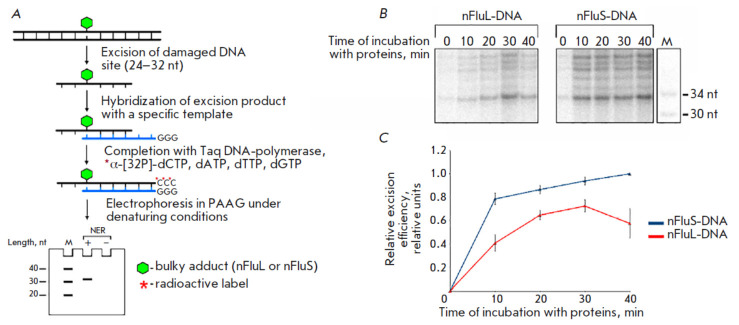
Comparative evaluation of the efficiency of removal of nFluL or nFluS from model DNA in a specific excision reaction catalyzed by the proteins of the HeLa cell extract. (*A*) is a schematic representation of the procedure for evaluating the excision activity of NER *in vitro *by the method of end labeling of specific excision products, adapted from [14, 23]; (*B*) is a radioautograph of the gel after the separation of excision reaction products; (*C*) is a graph of the dependence of the relative efficiency in the excision of the damaged DNA site on the incubation time with NER proteins. The values for the curves were calculated taking into account the signal intensity of products with a length of 34 nt

The effect of the structural differences between nFluL and nFluS on the efficiency of their removal from DNA substrates was assessed by the end-labeling method for excision products [14] (*Fig. 2A*). Extended DNA duplexes (137 bp) containing a non-nucleotide unit, either nFluL or nFluS, at an internal position (68th) of one strand were used as substrates for NER. Model DNA substrates were incubated at 30°C for 0–0 min with the HeLa cell extract (containing NER proteins). After the reaction was stopped, specific excision products (24–32-nucleotide- long DNA fragments containing the lesion) were hybridized with the template (*Table 1*, ODN-9), which is complementary to the damaged DNA region (*Fig. 2A*). The specific excision products hybridized to the template were extended with Taq DNA polymerase. The specific excision products that hybridized to the template were extended with Taq DNA polymerase and a mixture of dNTPs with [α-32P]dCTP, resulting in the labeling of the excision products. The extended excision products were predominantly 34 nucleotides long. Separation of the excision products was performed by denaturing PAGE (*Fig. 2B*). Based on the intensity of the 34-nucleotide bands, graphs of relative excision efficiency were plotted as a function of the incubation time of nFluS-DNA or nFluL- DNA with NER proteins (*Fig. 2C*).

As the results indicate, nFluS was removed from the model DNA 1.5–2 times more efficiently than nFluL during the entire experiment. Additional experiments allowed us to compare the changes — in dsDNA geometry and stability — induced by these bulky lesions; these changes influence the efficiency of DNA damage removal by the NER system.

**Geometrical Features of Model DNAs Containing the Bulky Modification nFluL or nFluS**

**Fig. 3:**
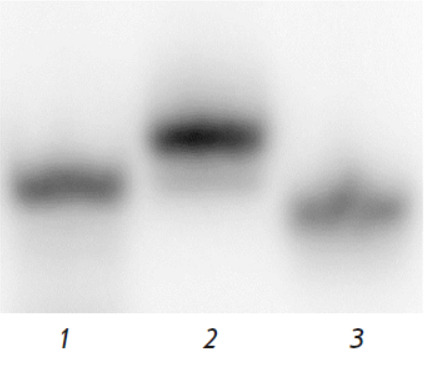
A comparison of electrophoretic mobility among 16- mer DNA duplexes. An autoradiograph of a polyacrylamide gel after electrophoresis under nondenaturing conditions. Lane *1 *– nFluS-dsDNA, lane* 2 *– nFluL-dsDNA, and lane *3 *– NM-dsDNA

One of the irregularities — in the regular dsDNA structure — recognized at the initial stage of damage recognition is a bending of the sugar-phosphate backbone at the lesion site. The bend magnitude depends on the type of lesion and the surrounding sequence [24]. Decreased compactness of the curved structure hinders DNA migration through polyacrylamide gel pores. The greater the backbone bend caused by a bulky modification, the lower the mobility of the duplex during nondenaturing PAGE. Accordingly, the geometrical features of 16-mer DNA duplexes carrying the nFluL or nFluS modification in one strand were determined based on their electrophoretic mobility in nondenaturing gels. A typical autoradiograph of the gel illustrating these experiments is shown in *Fig. 3*.

Judging from the calculated bending angles (see “Determination of the bending angle of DNA duplexes”), the nFluL-containing DNA duplex had suffered a stronger bend, with a bending angle of 165.40 ± 0.95°, consistent with earlier data [17, 25]. In contrast, the bending angle in nFluS-dsDNA was 170.64 ± 0.83°. These differences in backbone bending between the two substrates may be a result of dissimilar interactions of the fluorescein group with neighboring nucleotide bases. Molecular dynamics simulations suggest that the nFluL moiety is predominantly located outside the duplex, whereas substantial mobility in the damaged region results in frequent appearance/disappearance of a bend (~135°) [17]. Conversely, nFluS appears less prone to eversion, likely owing to the fluorescein group’s location within the duplex. Despite the smaller bending angle in nFluS-DNA, it was excised more efficiently by HeLa cell extract proteins. As shown previously [11], however, the efficiency of bulky-adduct repair does not always correlate with the degree of DNA helix bend.

**The comparison of the thermal stability of duplexes**

**Fig. 4:**
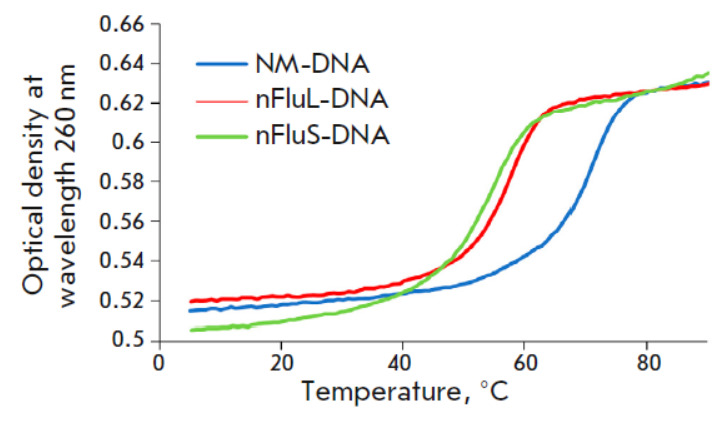
The dependence of the optical density of DNA duplexes at 260 nm on temperature

The thermal stability and thermodynamic characteristics of the 16 bp DNA duplexes were determined by thermal denaturation with optical signal recording. Analysis of the denaturation curves revealed that the hypochromic effect in the unmodified duplex was more pronounced as compared to duplexes containing a non-nucleotide linker. In particular, the hypochromic effect was 14.75% for NM-DNA, 13.5% for nFluL-DNA, and 14.3% for nFluS-DNA. Normalized graphs of optical density vs. temperature illustrate the differences in thermal stability among the duplexes (*Fig. 4*).

The unmodified DNA duplex was found to be the most thermally stable (Tm = 67.6 ± 0.3°C), while the nFluL duplex manifested Tm of 55.6 ± 0.3°C; and nFluS, the lowest thermal stability (Tm = 51.5 ± 0.3°C). Thus, nFluL and nFluS destabilize the duplex structure more than does another bulky lesion, nAnt (Tm for nAnt DNA = 59.6 ± 0.2°C) [16], with nFluS inducing the greatest destabilization. Other molecular modeling studies suggest that fluorescein groups (Flu-dU and nFluL) linked to the sugar-phosphate backbone by extended linkers disturb and destabilize the regular dsDNA structure through interactions with neighboring nucleotide bases. Molecular dynamics simulations have revealed that the fluorescein group of nFluL can frequently get everted from a duplex [17, 25].

Our data suggest that the much-reduced thermal stability of nFluS-containing duplexes, compared to nFluL, may be due to the preferential positioning of the nFluS fluorescein group within the duplex, where it distorts the DNA structure and interacts with nearby nucleotide bases [26].

**Fig. 5:**
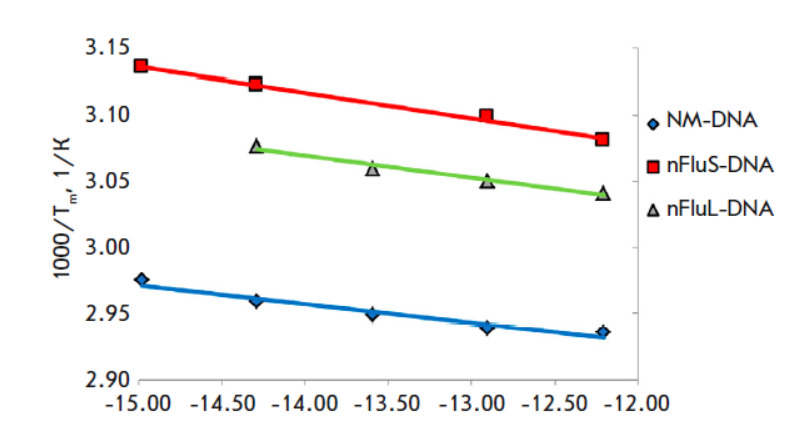
A linear van ‘t Hoff plot for the unmodified DNA duplex and duplexes containing an nFlu lesion (nFluL or nFluS)

Through minimization of the sum of squares of the deviations between the experimental and theoretical thermal denaturation curves, the thermodynamic parameters of melting were calculated next. ΔS° and ΔH° were determined by linearization of the expression in coordinates 1000/Tm and ln(Ct/4) (van ‘t Hoff coordinates) and construction of a linear dependence of the reciprocal of the melting temperature on the logarithm of the concentration ln(Ct/4) by the leastsquares method. The graphs are presented in *Fig. 5*. The results of the calculations of the thermodynamic parameters in *Table 2* also indicate an enhanced ability of nFluS to destabilize the double-stranded structure of surrounding DNA as compared to nFluL.

**Table 2:** Thermodynamic data and the melting temperatures of the DNA duplexes

DNA duplex	ΔS°, cal/(mol×K)	ΔH°, kcal/mol	ΔG°37, kcal/mol	Tm (20 μM), °C
NM-DNA	-382 ± 167	-140.0 ± 56.7	-20.2 ± 4.8	67.6 ± 0.3**
nFluL-DNA	-339 ± 187	-119.4 ± 61.2	-14.3 ± 3.2	55.6 ± 0.3**
nFluS-DNA	-295 ± 104	-103.5 ± 33.5	-12.0 ± 1.2	51.5 ± 0.3**

^**^Standard deviation for temperature takes into account the error of the measuring instrument.

**Spectrometric titration**

**Fig. 6:**
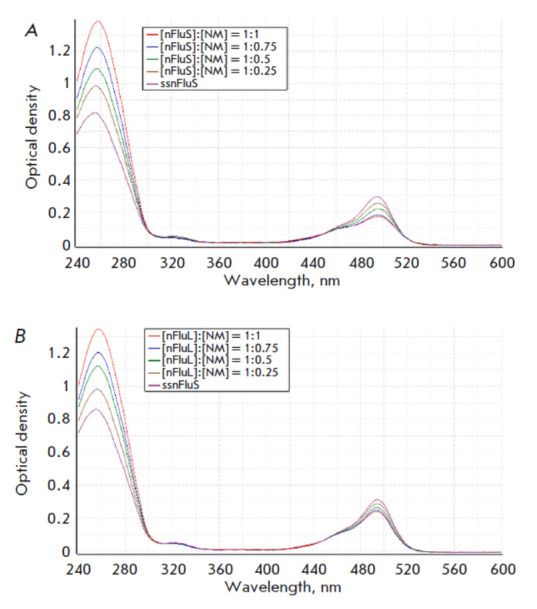
Optical absorption spectra of samples containing nFluS-DNA (*A*) or nFluL-DNA (*B*) at a constant concentration of 5.4 μM. Concentration ratios are shown for samples containing modified (nFluS- or nFluL) and unmodified (nm) strands that form duplexes

The spectrometric titration method, in combination with other approaches, allows one to draw conclusions about the presence of a number of interactions involving (or not involving) a fluorescent group on the basis of data on the changes in the spectral characteristics caused by duplex formation. The most noticeable differences between the two lesions containing a bulky carboxyfluorescein moiety were revealed in a comparative analysis of DNA duplexes containing the bulky modification nFluL or nFluS by spectrometric titration. The samples contained a modified nFluS or nFluL strand at a fixed concentration of 5.4 μM; the concentration of complementary DNA varied. The control samples in each series contained only the modified strand. The measurements were carried out after hybridization of the strands. The optical absorption spectra are given in *Fig. 6*.

At 260 nm, a gradual increase in the optical density of both series of samples was observed, the result of an increase in the total concentration of DNA in the mixture. By contrast, at 495 nm (the maximum absorption of fluorescein), the nature of the change in the optical density of the samples containing nFluS-DNA or nFluL-DNA changed as the concentration of the complementary strand in the samples went up, and, consequently, the amount of double-stranded structures rose too.

A comparison of optical absorption between nFluS-DNA and nFluL-DNA via titration of unmodified DNA was performed next. It showed that in the region of maximum nucleotide absorption (260 nm) in both DNA series, along with an increase in the optical density, an identical hypochromic effect was present, all due to the formation of duplexes (*Fig. 6*). The nature of the changes in the optical density near the maximum fluorescein absorption (495 nm) differed: in the case of DNA duplexes containing nFluS, the hypochromic effect, which appeared at the maximum concentration of the intact strand, amounted to 40%, whereas in DNA duplexes containing nFluL, it did not exceed 20% (*Fig. 6*).

**Fig. 7:**
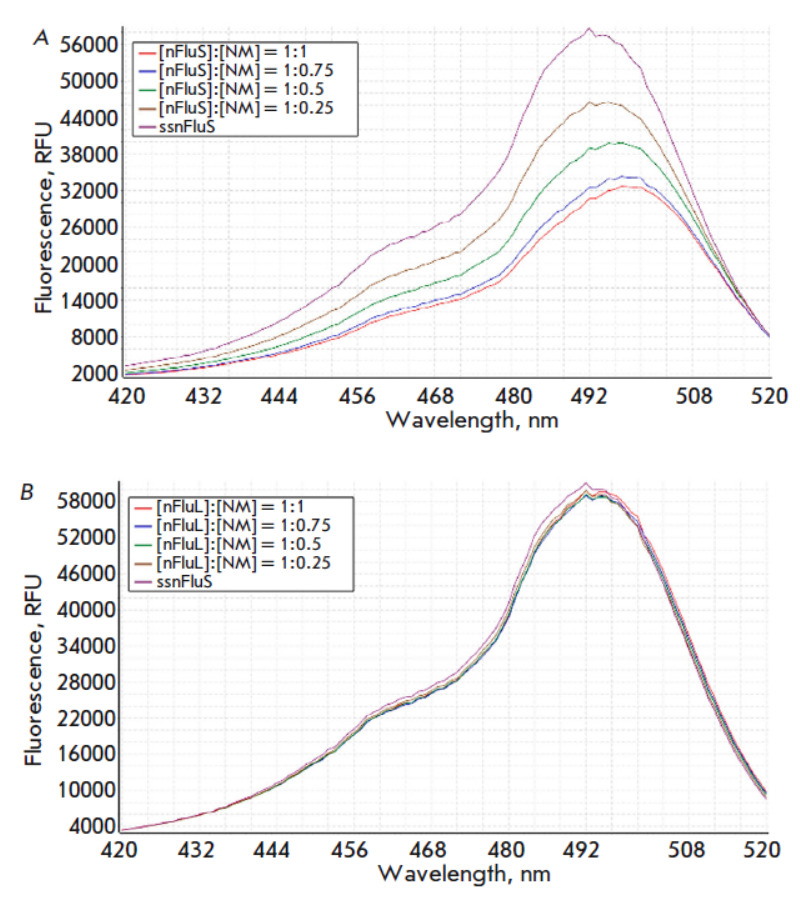
Fluorescence excitation spectra in the range of 420–520 nm in a series of samples containing nFluS-DNA (*A*) or nFluL-DNA (*B*) at a concentration of 5.4 μM and a complementary strand without modifications (nm)

Elsewhere, using the absorption spectra of DNA duplexes containing *cis*- and *trans*-B[α]P-N2-dG lesions as an example, it has been demonstrated that the formation of duplexes in which the benzo[α]pyrene (B[α]P) adduct forms strong intercalation complexes is accompanied by a hypochromic effect manifesting itself in the absorption region of the pyrenyl rings of B[α]P [27]. Thus, the results of our spectrophotometric titration showed that the presence of a lesion containing a longer spacer moiety leads to a level of local DNA destabilization sufficient for the efficient recognition and removal of nFluL by NER proteins; however, its fluoresceinylcarbamoyl group does not form stable sandwich structures or any other complexes the formation of which causes noticeable changes in the spectrum. These findings are in agreement with recently-performed molecular dynamics simulations of nFuL-containing DNA trajectories [17].

**Fig. 8:**
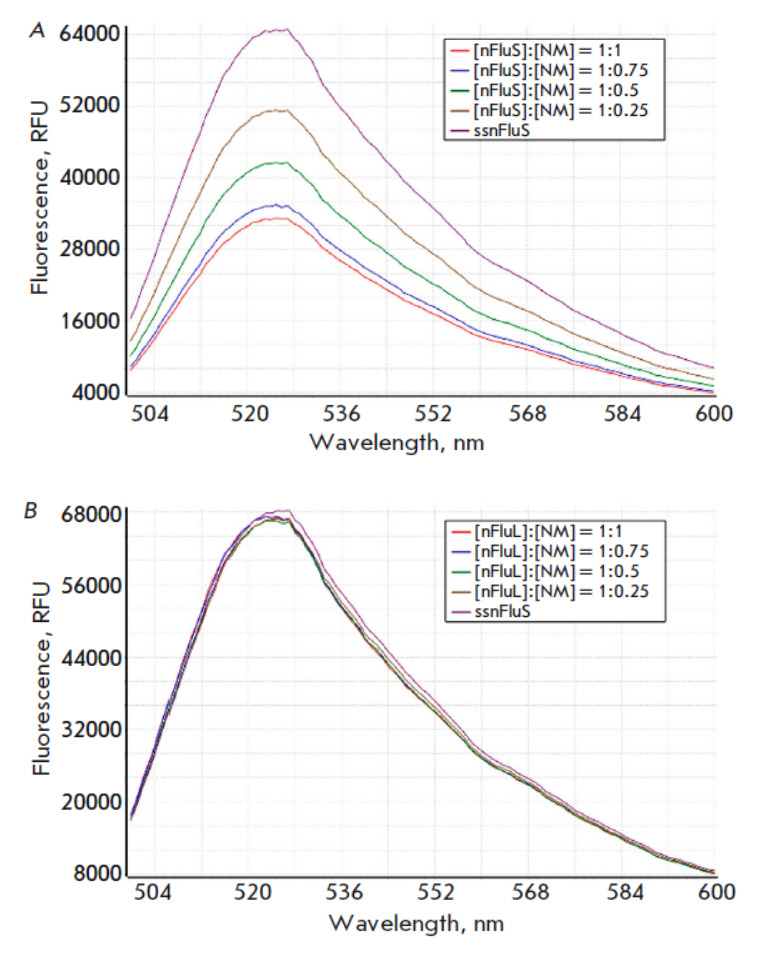
Fluorescence emission spectra of the series of samples containing nFluS (*A*) or nFluL (*B*) at 5.4 μM and the complementary strand without modifications (nm)

The most illustrative results, allowing us to determine the reasons for the differences in properties between the two fluorescein-based lesions, were obtained in a comparison of fluorescence spectra between the two series of samples containing nFluS-DNA or nFluL-DNA and the unmodified DNA strand at the same ratios as in the analyses of changes in the optical spectra. The resultant excitation and emission spectra of DNA duplexes containing the nFluS or nFluL lesion are shown in *Fig. 7* and *Fig. 8*, respectively. The fluorescence excitation spectra were recorded at a fixed emission wavelength of 548 nm; the excitation wavelength was varied in the range of 420–520 nm with a step of 1 nm (*Fig. 7A,B*, respectively). Emission spectra — representing the dependence of the light emission intensity of the fluorophore (Flu) on the wave length of the excitation light — were recorded in the range of 500–600 nm with a measurement step of 1 nm at a fixed excitation wavelength of 533 nm (*Fig. 8A,B*). The fluorescence intensities of the samples containing nFluS-DNA noticeably and steadily decreased with an increase in the concentration of the complementary strand, whereas in the case of samples containing nFluL-DNA, which has an extended linker, the fluorescence intensity remained virtually unchanged (*Fig. 7A* and *Fig. 8A*).

Thus, the results of a spectrometric titration enable us to state with a high degree of confidence that, during duplex formation, the fluoresceinylcarbamoyl moiety of the nFluS lesion is located inside the duplex and remains capable of interacting with the guanosines of the DNA nucleotides closest to the lesion, similarly to the descriptions of some types of fluorescein positioning in DNA duplexes [26].

## CONCLUSION

We hypothesized that nFluS (N-[6-{5(6)-fluoresceinylcarbamoyl}]- 3-amino-1,2-propanediol) represents an analog of a bulky lesion whose efficiency of specific excision from model DNA is higher than that of nFluL (N-[6-{5(6)-fluoresceinyl carbamoyl}hexanoyl]-3-amino- 1,2-propanediol). The hypothesis was proven successfully. Comparative analyses of the geometrical features, thermodynamic characteristics, optical absorption spectra, and fluorescence spectra of model DNA duplexes containing nFluS or nFluL showed that the higher efficiency in the specific excision of nFluS compared to nFluL is explained by the additional destabilization of the dsDNA structure under the influence of the interaction of the fluoresceinylcarbamoyl moiety with neighboring nitrogenous bases of dsDNA. A given localization of the destabilized region in nFluS-containing dsDNAs probably promotes the formation of productive XPC–DNA complexes, thereby improving the substrate properties of nFluS-DNA in a specific excision reaction. Molecular modeling experiments are required to prove this interpretation right.

## Abbreviations

NER: nucleotide excision repair
GG-NER: global genome NER
nFluL: N-[6-{5(6)- fluoresceinylcarbamoyl}hexanoyl]-3-amino-1,2-propanediol
nFluS: N-[6-{5(6)-fluoresceinylcarbamoyl}]-3- amino-1,2-propanediol
ODN: oligodeoxyribonucleotide
